# Flexible topographical design of light-emitting diodes realizing electrically controllable multi-wavelength spectra

**DOI:** 10.1038/s41598-023-39791-2

**Published:** 2023-08-04

**Authors:** Yoshinobu Matsuda, Ryunosuke Umemoto, Mitsuru Funato, Yoichi Kawakami

**Affiliations:** Department of Electronic Science and Engineering, Kyoto University, Kyoto, Kyoto 615-8510 Japan

**Keywords:** Electrical and electronic engineering, Inorganic LEDs

## Abstract

Multi-wavelength visible light emitters play a crucial role in current solid-state lighting. Although they can be realized by combining semiconductor light-emitting diodes (LEDs) and phosphors or by assembling multiple LED chips with different wavelengths, these design approaches suffer from phosphor-related issues or complex assembly processes. These challenges are significant drawbacks for emerging applications such as visible light communication and micro-LED displays. Herein we present a platform for tailored emission wavelength integration on a single chip utilizing epitaxial growth on flexibly-designed three-dimensional topographies. This approach spontaneously arranges the local emission wavelengths of InGaN-based LED structures through the local In composition variations. As a result, we demonstrate monolithic integration of three different emission colors (violet, blue, and green) on a single chip. Furthermore, we achieve flexible spectral control via independent electrical control of each component. Our integration scheme opens the possibility for tailored spectral control in an arbitrary spectral range through monolithic multi-wavelength LEDs.

## Introduction

Impact of monolithic integration of electrical components such as transistors, diodes, and resistors into a single and compact chip has been significant in the field of electronics. Compared to discrete components, large-scale integration (LSI) technology offers improved performance, reduced cost, and increased reliability. Today, LSI technology is a cornerstone of modern electronics. However, while discrete monochromatic light-emitting diodes (LEDs), including InGaN-based blue and green LEDs and AlGaInP-based red LEDs, have been developed in the field of visible light optoelectronics, the monolithic integration of multiple wavelengths remains a challenge.

Two alternative options already exist for multi-wavelength light emitters for visible light optoelectronics. The most widely used method to date is to combine a blue InGaN LED with a yellow phosphor to produce a white emitter^1^. This construction allows a simple device configuration, but, at the same time, inevitably induces Stokes energy loss due to the color conversion from blue to yellow. In addition, independent electrical control of the phosphor emission is difficult, limiting the tunability of the emission spectra. Another commercial option to avoid the phosphor-related issues involves assembling red, green, and blue (RGB) LED chips, which can deliver a high degree of control over the overall color. However, this approach requires a complex and time-consuming assembly process and carefully designed external optics to ensure good color mixing.

These issues become more severe in emerging applications using visible light emitters. For instance, visible light communications^2^ and their extension to fully networked systems, which are referred to as Li-Fi^3^, have gained significant interest in the field of optical wireless communications, where white LEDs are used for both illumination and data communication. In optical communications, the slow response of yellow phosphors hampers higher modulation bandwidths. Additionally, communication capacity can be increased by wavelength division multiplexing (WDM) using multiple LEDs^2^. However, WDM in visible light communication tends to be limited to only three colors using separate RGB LEDs, despite the broad visible spectral range (380–780 nm). To further increase the number of data streams, more separate LEDs with different wavelengths must be fabricated and assembled into a single device. Meanwhile, micro LEDs ($\mu$LEDs) with the size of less than $\sim$ 100 $\times$ 100 $\upmu$m$^2$ are promising for display applications due to several potential advantages such as high contrast, fast response, and high efficiency, compared to conventional liquid crystal displays and organic LED displays^4,5^. One challenge for mass production is the precise transfer of millions of individual LED dies onto the backplane, and considerable research efforts have been devoted to developing the transfer technologies^4^. To radically address these issues, solutions for monolithic integration of multiple wavelengths on a single substrate are necessary.

In principle, the wide tunability of the InGaN bandgap can cover the entire visible spectral range^6^, suggesting that visible light can be integrated on one chip via a single epitaxial growth process. Recently, various approaches have been intensively studied toward tailored multiple-wavelength integration. One promising approach is three-dimensional (3D) GaN structures formed by selective area growth (SAG) technique. Regrowth of GaN on patterned Ti masks by molecular beam epitaxy creates nanocolumn (NC) structures^7–10^. The emission wavelengths of the NC LEDs are controlled by the NC diameters, and monolithic integration of multicolor micro-LED pixel units has been demonstrated^10^. However, the 3D geometry with high aspect ratios increases the process complexity. Another approach is regrowth of GaN on patterned dielectric masks by metal-organic vapor phase epitaxy (MOVPE), which creates 3D structures composed of several crystallographic facets^11–22^. The facet-dependent emission wavelength allows pastel color syntheses, including white^14–17,21,22^, whereas the facets formed by SAG are restricted to stable crystallographic planes during growth, limiting flexible wavelength integration.

Herein, we present monolithic multi-wavelength InGaN LEDs based on flexibly-designed 3D topographies. Topography patterning on the substrate surfaces spontaneously arranges the emission wavelengths of the overgrown InGaN LED structures through the local In composition variations^23–29^. A key feature of this approach is that not only stable planes exposed in the 3D structures by SAG but also unstable planes can be used, thus providing greater flexible wavelength integration. In addition, the emission wavelengths can be widely controlled within a shallow slope angle ($<\ \sim$ 10$^\circ$)^23–28^, which is beneficial for device processing^29^. We demonstrate monolithic multi-wavelength InGaN LEDs, which can electrically control each emission component independently. The single LED chip provides three different colors of violet, blue, and green, and the emission components are flexibly synthesized under independent operations.

## Integration scheme and device structure

**Figure 1:**
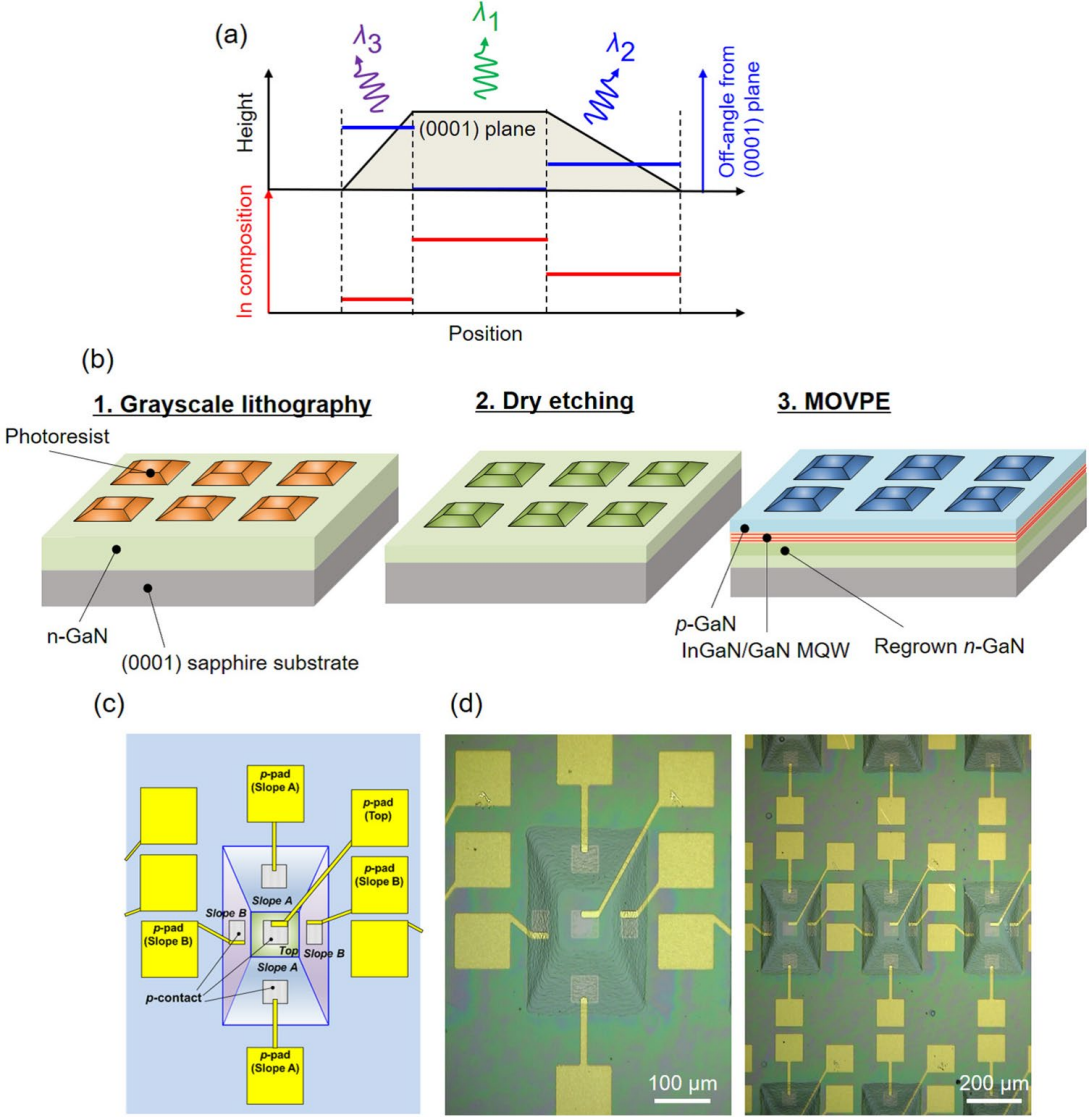
Integration scheme of the local off-angles and fabrication procedure. (**a**) Schematic of local off-angle integration, including an example of a cross-sectional height profile formed on a (0001) GaN surface and the corresponding off-angle profile from the (0001) plane. Expected In composition distribution of the overgrown InGaN QW is also shown. (**b**) Fabrication process of monolithic multi-wavelength InGaN LEDs on polyhedral structures. (**c**) Schematic of the device design with the three different emission wavelengths used in this study and (**d**) the optical microscope images of a single device (left) and an array (right).

Figure 1a illustrates the integration scheme of the three emission wavelengths in this study. Due to crystal anisotropy, tilting the surface orientation from the (0001) plane drastically changes the growth behavior. In particular, In incorporation during InGaN quantum well (QW) growth for the light-emitting layers initially decreases as the off-angle from the (0001) plane increases. Because this shortens the emission wavelengths of the InGaN LEDs^23–28^, the emission wavelengths from the three parts with different off-angles in Fig. 1a decrease in the order of $\lambda _1>\lambda _2>\lambda _3$. The number of integrated wavelengths is equivalent to the number of integrated off-angles. The local off-angles are patterned on the (0001) GaN surfaces using the following procedures.

The LED structure with local off-angle patterning was fabricated by grayscale lithography and dry etching^27^, followed by MOVPE (Fig. 1b) (for details see “Methods”). The structure was fabricated on (0001) n-GaN/sapphire templates. To demonstrate local off-angle integration, a photoresist polyhedral shape was designed. The tilt angles of the sloped faces in the polyhedral shape determined the off-angle with respect to the (0001) plane (*i*.*e*., larger off-angles with steeper slopes). First, polyhedral photoresists were formed on the GaN surfaces by grayscale lithography. The polyhedral shape of the photoresist was subsequently transferred to the GaN surfaces using inductively coupled plasma reactive ion etching (ICP-RIE). Then InGaN-based LED structures composed of n-GaN, InGaN/GaN multiple QWs (MQWs), and p-GaN layers were regrown by MOVPE.

Figure 1c schematically depicts the LED design used in this study. The polyhedral structure consists of a planar top part (Top) and two sloped parts (Slope A and Slope B). The opposite slopes are congruent for simplicity. The in-plane dimension of the 3D shape is about 220 $\upmu$m $\times$ 340 $\upmu$m, which is comparable to that of a conventional planar LED (300 $\times$ 300–500 $\times$ 500 $\upmu$m$^2$). Slope A has a smaller off-angle than that of Slope B, as shown below. This design varies the emission wavelengths in the order of Top > Slope A > Slope B. Thanks to the gently-sloping 3D structures (Supplementary Note 1), standard binary photolithography and vacuum evaporation, which are commonly used for planar LEDs, were used to fabricate the LED device. The p-contact electrodes were separately formed on each part of the polyhedral structure and connected to p-pad electrodes for probing. N-electrodes were formed on the sample edge. The final device was on-wafer without packaging. Figure 1d displays optical microscope images of the fabricated InGaN LEDs, confirming successful electrode formation. It should be noted that the array of the multi-wavelength InGaN LEDs can be applied to spontaneous arrangement of the $\mu$LED pixel units for display applications.

## 3D shape and local emission characteristics

**Figure 2:**
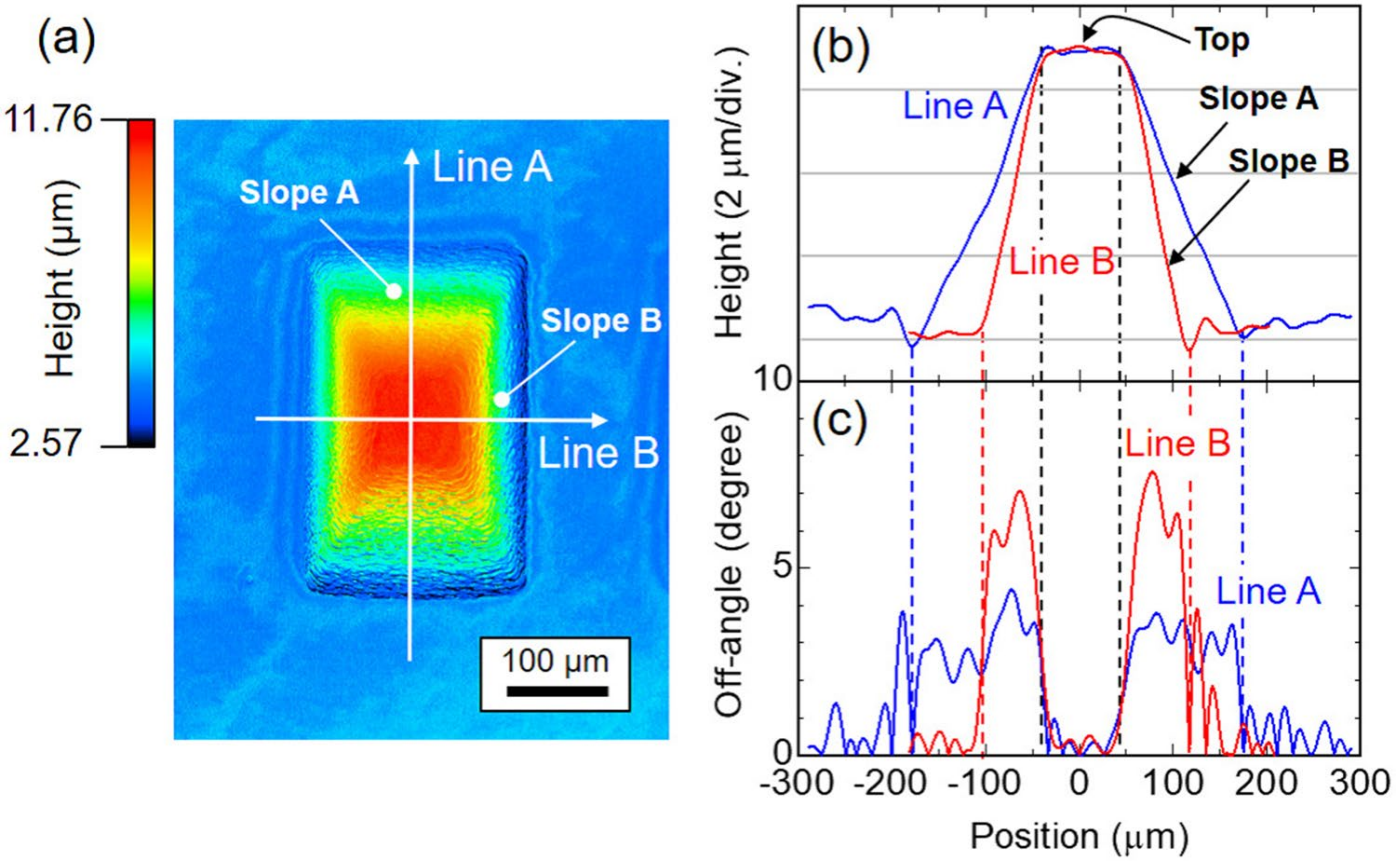
3D shape characterization of the InGaN QW on the polyhedral structure. Epi-layer consists of n-GaN and InGaN SQW without p-GaN. (**a**) Height mapping image. (**b**) Cross-sectional height profiles along the lines indicated in (**a**), and (**c**) off-angle profiles calculated from (**b**).

Prior to LED device operations, the relationship between the 3D shape and local emission characteristics was investigated. Figure 2 displays the evaluation results of a 3D shape of an InGaN QW on the polyhedral structure using confocal laser scanning microscopy. The epi-layer consists of n-GaN and an InGaN single QW (SQW) without p-GaN (see “Methods”). The height mapping image reveals a truncated square pyramid structure (Fig. 2a). Figure 2b shows the extracted cross-sectional height profiles along the lines indicated in Fig. 2a. Slope A has a shallower slope than that of Slope B. The off-angle profiles calculated from Fig. 2b are shown in Fig. 2c, where the off-angle is defined with respect to the (0001) plane. That Slope A has a smaller off-angle than that of Slope B is clearly confirmed. The off-angle variation in each slope is due to the surface roughness formed during the regrowth process (Supplementary Note 2), and improving the uniformity is a subject for future research.

**Figure 3:**
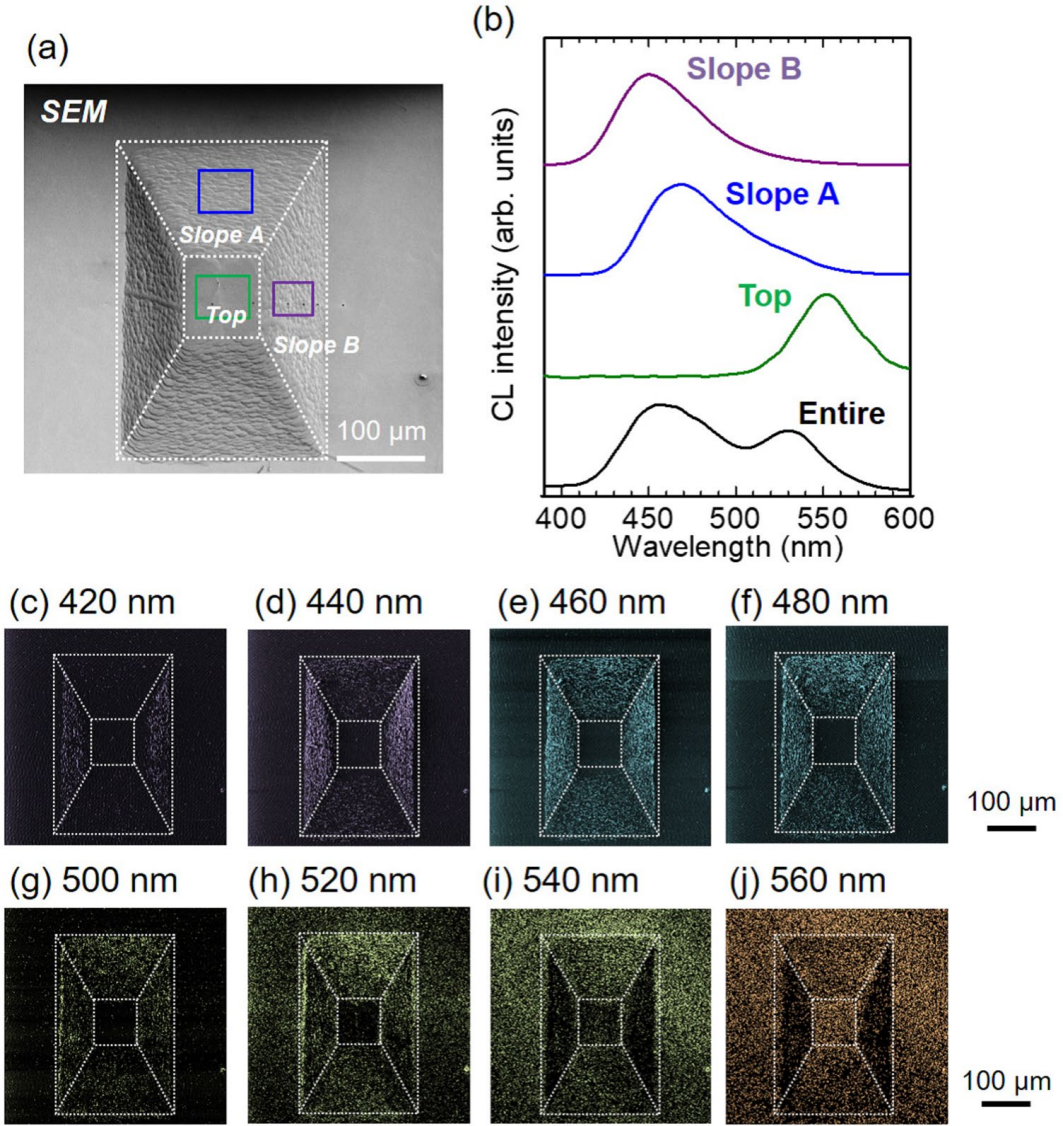
Local emission characteristics of the InGaN QW on the polyhedral structure. (**a**) Top view SEM image. Dotted lines are to guide the eye. (**b**) CL spectra acquired from the entire area and from the magnified areas indicated as boxes in (**a**). Monochromatic CL mapping images acquired at (**c**) 420 nm, (**d**) 440 nm. (**e**) 460 nm, (**f**) 480 nm, (**g**) 500 nm, (**h**) 520 nm, (**i**) 540 nm, and (**j**) 560 nm.

Figure 3 summarizes the spatially resolved emission properties of the InGaN QW evaluated by scanning electron microscopy (SEM) and cathodoluminescence (CL) spectroscopy at room temperature (RT). Figure 3a shows the top-view SEM image of the InGaN QW, and Fig. 3b depicts the CL spectra acquired from the entire area and the magnified areas of Top, Slope A, and Slope B, indicated in Fig. 3a. The dimensions of the magnified areas approximately correspond to those of each p-contact electrode. The peak wavelengths are located at 552 nm on Top, 469 nm on Slope A, and 450 nm on Slope B. Considering the 3D shape (Fig. 2), the emission wavelength decreases as the off-angle increases, in accordance with the previous reports^23–29^. Supplementary Note 3 details the structural properties of the InGaN QWs assessed by transmission electron microscopy. The monochromatic CL mapping images in Fig. 3c–j reveal a position dependence of the emission wavelength. As the monitoring wavelength becomes longer, the light emission begins in the order of Slope B, Slope A, and Top. The obtained results demonstrate that the polyhedral shape can integrate three emission wavelengths through local off-angle control.

## Fundamental device performance and additive spectral mixing

**Figure 4:**
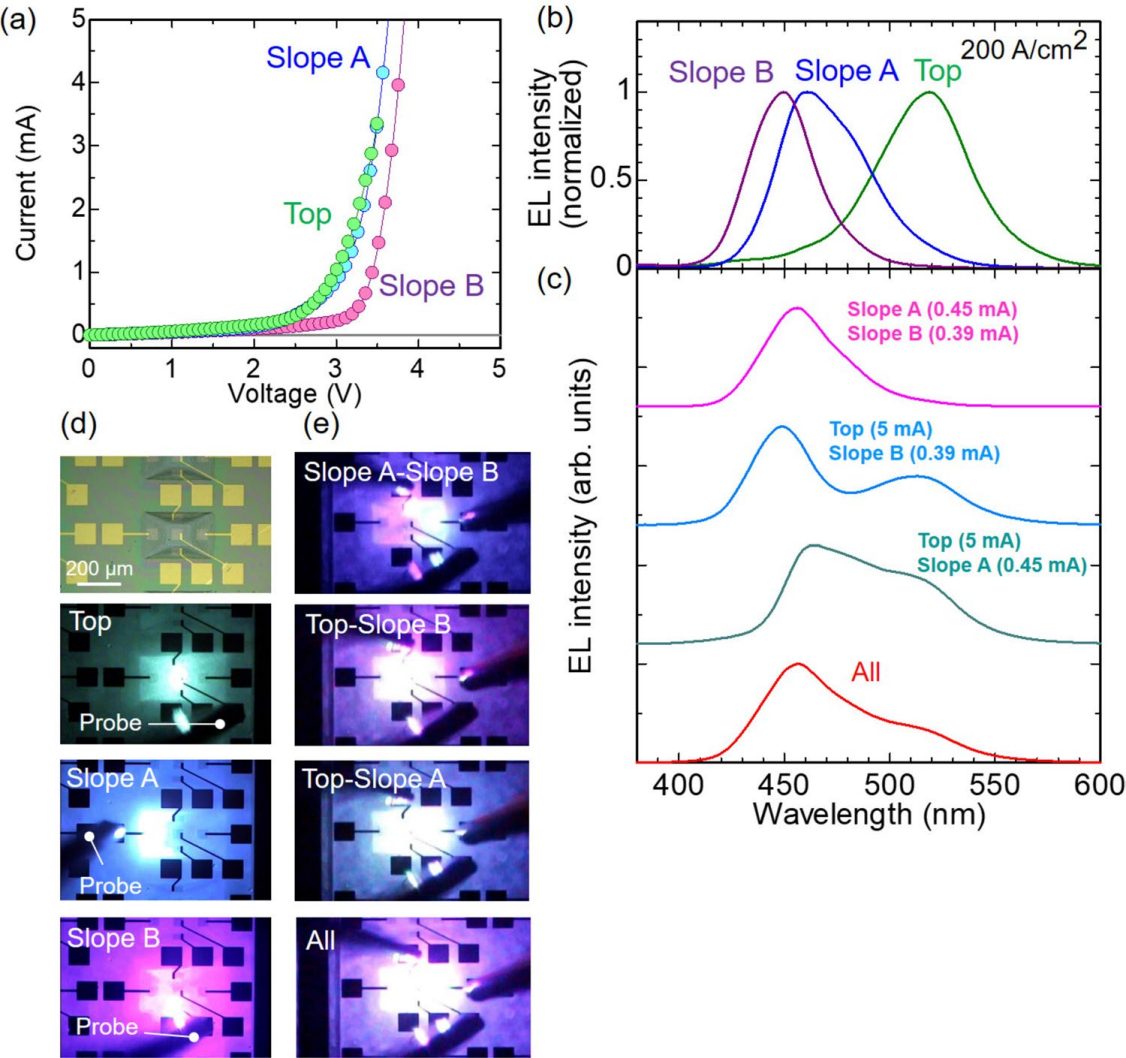
Fundamental device performance and additive spectral mixing of the monolithic multi-wavelength LED. (**a**) Current–voltage characteristics of InGaN LEDs formed on Top, Slope A, and Slope B. (**b**) Normalized EL spectra of the three LEDs individually driven at an injection current density of 200 A/cm$^2$. (**c**) EL spectra under simultaneous operations of two or all three of the LEDs. Injection currents are 5 mA at Top, 0.45 mA at Slope A, and 0.39 mA at Slope B. Peak EL intensities from the three parts are almost the same under this condition. Optical microscope images of (**d**) the individual operations in (**b**), and (**e**) the simultaneous operations in (**c**). Topmost image in (**d**) is the sample not operated to clearly indicate the measurement locations.

We separately evaluated the device performance of the InGaN LEDs formed on Top, Slope A, and Slope B in a single polyhedral structure (single chip). Figure 4a shows the current−voltage characteristics of the three LEDs driven under a direct current (DC) injection. The forward characteristics of the diodes are confirmed. The estimated turn-on voltages and series resistances are about 3.0 V and 165 $\Omega$ for Top, 3.3 V and 61 $\Omega$ for Slope A, and 3.6 V and 55 $\Omega$ for Slope B. The higher series resistance at Top can be attributed to the larger p-GaN thickness or lower carrier density. Higher hole carrier densities with larger off-angles from the (0001) plane are reported^34^. Typical electroluminscence (EL) spectra under an injection current density of 200 A/cm$^2$ (5 mA for Top and Slope A and 3 mA for Slope B) are displayed in Fig. 4b. The peak wavelengths are 519 nm at Top, 460 nm at Slope A, and 450 nm at Slope B, which are consistent with the CL spectra (Fig. 3b). The EL peaks of Top and Slope A are located at shorter wavelengths than those of the CL peaks, and the difference increases as the emission wavelength becomes longer. InGaN-based LEDs often exhibit a blueshift in the emission wavelength as the injection current density increases, and the peak wavelength shift is more pronounced for longer emission wavelengths. Thus, the relatively high current density (200 A/cm$^2$) under the EL conditions may cause the peak wavelength difference between EL and CL. The optical microscope images in Fig. 4d confirm the integration of the three different colors in the single chip: green from Top, blue from Slope A, and violet from Slope B.

We then demonstrate the simultaneous operations of the LEDs on Top, Slope A, and Slope B. Figure 4c shows the EL spectra when two or all three of the LEDs are operated simultaneously. To highlight spectral mixing, the injection current for each LED is adjusted to produce comparable EL intensities. In particular, the simultaneous operations of Top and Slope A or Slope B clearly show the synthesis of shorter and longer wavelength components, confirming additive spectral mixing. The mixed colors change due to the wavelength difference of Slopes A and B (Fig. 4e): bluish or purplish pastel colors are synthesized by adding Slope A or Slope B to Top, respectively. Therefore, the proposed LED design enables to provide three different colors individually and further allows the synthesis of these components to meet specific requirements. The Supplementary video shows individual and simultaneous operations. Although this study manipulates the injection currents to control the EL intensities, pulse-width modulation used in LED lighting should be possible. The results will be reported elsewhere.

## Flexible spectral control

**Figure 5:**
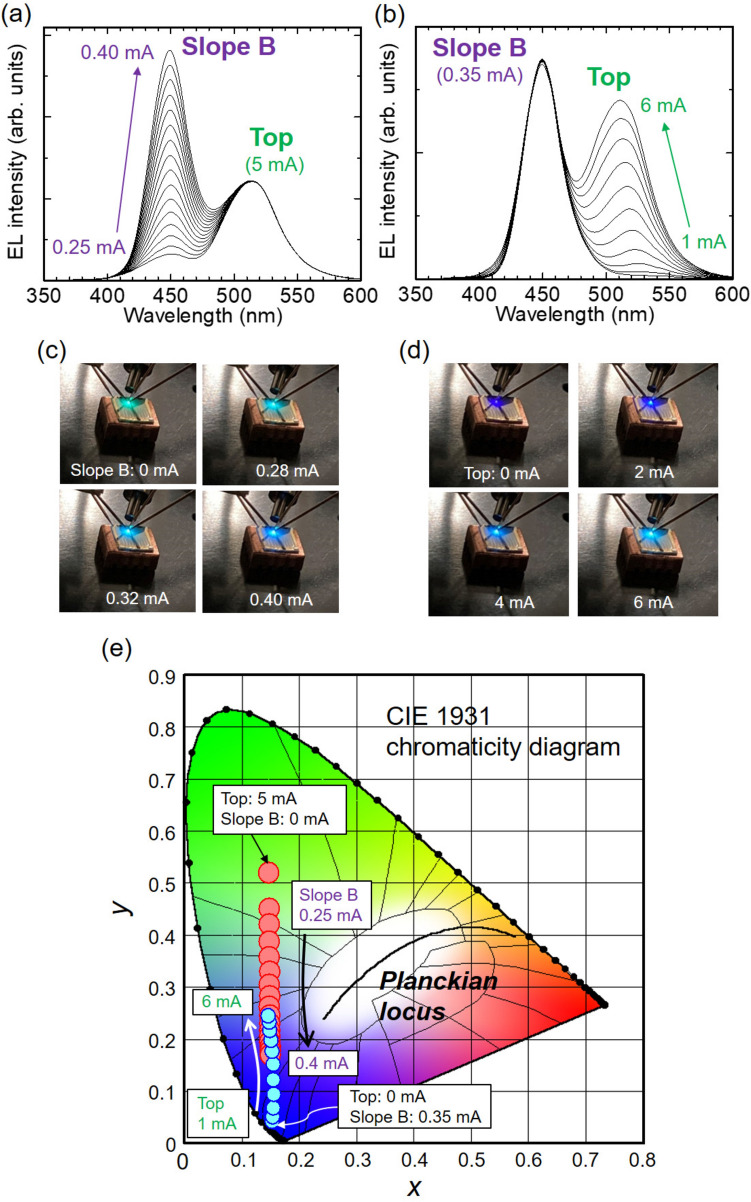
Flexible spectral control using the single LED chip. EL spectra variation under injection currents of (**a**) 5 mA at Top and 0.25–0.40 mA at Slope B and (**b**) 1–6 mA at Top and 0.35 mA at Slope B. (**c**,**d**) The photographs under operations of (**a**,**b**), respectively. (**e**) CIE chromaticity diagram for the spectral controls.

Finally, we demonstrate flexible spectral control by electrically tuning the LEDs. As a representative case, we present the results using a combination of the LEDs on Top and Slope B. Figure 5a,b show the EL spectra under different injection current combinations: (Top, Slope B) = (5 mA, 0.25–0.40 mA) and (1–6 mA, 0.35 mA), respectively. In Fig. 5a, only the intensity of the shorter wavelength component from Slope B is varied, while only that of the longer wavelength component from Top is varied in Fig. 5b. These results show that the apparent color can be adjusted from green to pastel blue or from violet to pastel blue, as shown in Fig. 5c,d, respectively (Supplementary video). Figure 5e plots the emission color variations on the Commission Internationale de I’Éclairage (CIE) 1931 chromaticity diagram. Increasing the injection current to Slope B varies the emission color from (0.15, 0.52) to (0.15, 0.17), while increasing the injection current to Top varies the emission color from (0.16, 0.04) to (0.15, 0.24).

## Discussion

We discuss several topics as future research subjects to further improve our strategy. The first subject is optical and current crosstalk in the proposed LEDs. Optical crosstalk between each LED may lead to lower pixel contrast ratios in display applications. Additionally, short-wavelength LEDs can excite the long-wavelength LEDs, degrading the color purity. Experimentally, Figs. 4b, and 5a,b reveal that the blue or violet emissions from Slope A or B barely excite the green emission from Top. To surely suppress the optical crosstalk, black matrix photoresists inserted between each LED, which have almost no transmittance in the visible spectral range, would be useful^35^. Current crosstalk between each LED is also possible in the proposed LEDs because the p-GaN isolation was not employed in this study (see “Methods”). However, we note that the distances between each LED are approximately 40–70 $\upmu$m (Fig. 1d), and the lateral current spreading in the p-GaN is estimated to be on the order of a few micrometers by using a simple electrical model^36^. Additionally, as shown in Fig. 5a,b, the emission intensities from the LEDs [Top in Fig. 5a or Slope B in Fig. 5b] driven under the constant current do not change with varying the current into the other LEDs [Slope B in Fig. 5a or Top in Fig. 5b]. These results suggest that the current crosstalk in the proposed LEDs would be negligible. If the distance between each LED approaches the order of a few micrometers, it will be necessary to isolate the p-GaN through dry etching or selective passivation^37^.

The second subject is to realize high-quality InGaN LEDs on different surface orientations using a single epitaxial growth process. The optimum growth conditions may vary depending on the surface orientations. However, at present, the relationship between optimum growth conditions of InGaN and surface orientations has not been extensively studied. Therefore, clarifying the growth behavior and consequent emission efficiency of InGaN on various surface orientations is an important issue, and our patterned templates can serve as a platform. It is expected that the differences in optimum growth conditions due to surface orientation would not be so significant, given that the proposed structures exhibit the large wavelength variation (450–552 nm) (Fig. 3b) within a relatively narrow off-angle range (< 10$^\circ$) (Fig. 2c).

The third subject is to expand the range of emission wavelengths toward longer wavelengths. The emission efficiency of InGaN-based LEDs tends to decrease in the range of longer wavelength components such as yellow and red. However, recent intensive researches are pushing the emission wavelength longer, and the emission efficiencies of yellow and red InGaN LEDs are steadily improving by exploring the growth conditions or stacking layers^30,31^.

The fourth subject is to increase the number of controlled wavelengths. A straightforward approach based on our results is to increase the number of faces with different slope angles in the polyhedral structure. Alternatively, we have already proposed using convex lens-like structures to achieve continuously changing off-angles^28,29^. This type of structure has the potential to offer an immense number of wavelengths due to the continuously changing wavelength distribution. Increasing the number of integrated wavelengths could complicate the electrode design. However, we note that the accuracy of device processes is not related to pattern complexity but to pattern size comparable to process resolutions. Moreover, conventional process conditions similar to those for planar LEDs can be applied to the proposed LEDs with shallow slope angles (see “Methods”). Therefore, the proposed LED design could increase the number of integrated wavelengths without complex process optimization.

Finally, we would like to note that the proposed LEDs can be applied to multi-wavelength photodetectors. Conventional photodetectors measure an intensity of light at a specific wavelength dispersed by a spectrometer. In contrast, these multi-wavelength photodetectors potentially offer the ability to selectively measure intensities at multiple wavelengths without the spectrometer.

## Conclusion

In conclusion, monolithic multi-wavelength InGaN LEDs are fabricated using local off-angle integration. Our approach allows multiple emission wavelengths to be spontaneously arranged on a single LED chip. The resultant device realizes flexible electrical control of violet, blue, and green emission colors, and can synthesize these components to meet specific requirements. Our achievements have the potential to advance the development of tailored visible spectral syntheses based on monolithic multi-wavelength InGaN LEDs. Visible light emitters with flexible spectral designability should benefit numerous applications such as advanced solid-state lighting, high capacity communications by WDM in Li-Fi systems, and spontaneously arrayed RGB $\mu$LEDs for display applications.

## Methods

### Substrate patterning

The local off-angle distribution was patterned on (0001) n-GaN/sapphire templates with about 13-$\mu$m thick n-GaN. First, polyhedral photoresist shapes were formed by a grayscale lithography technique using mask-less exposure equipment (Nano System Solutions, D-light DL-1000GS/KCH). Grayscale lithography creates 3D micro-structures with height gradients in a positive photoresist^32,33^. Intensity gradients of the exposure light are converted into exposure depth and subsequently into resist topography on the micro-scale. The spin-coated photoresist (TOKYO OHKA KOGYO, PMER P-LA900PM) had a designed thickness of $\sim$8 $\mu$m. After shaping the 3D photoresists, the photoresist surface was smoothed using a thermal reflow method on a hotplate at 110 $^\circ$C for 3 minutes. The 3D shape of the photoresists was transferred to the GaN surface by ICP-RIE (Samco, RIE-200KNS) using Cl$_2$ gas with ICP and bias powers of 400 W and 300 W, respectively. The residual photoresist was removed using sulfuric acid-hydrogen peroxide mixtures.

### Crystal growth and device fabrication

InGaN LED structures were regrown on the patterned substrates by MOVPE (Taiyo Nippon Sanso, SR2000). The LED structure consisted of Si-doped n-GaN (500 nm), InGaN (2.5 nm)/GaN (11 nm) three-period QWs, Mg-doped p-GaN (300 nm), and heavily Mg-doped p$^+$-GaN (20 nm) layers. The thickness values represent those on the planar substrate without topography patterning. To accurately relate the local off-angle distribution and the local emission properties of the InGaN QWs, we also fabricated a structure composed of n-GaN and InGaN single QWs without a p-GaN layer. This is because the p-GaN cap layers can alter the 3D shape^29^. The source precursors were trimethylgallium for n-GaN and p-GaN, triethylgallium and trimethylindium for InGaN/GaN QWs, and ammonia. Si and Mg dopant sources were silane and bis(cyclopentadienyl)-magnesium, respectively. The growth pressure was 100 kPa throughout the entire growth sequence, while the growth temperatures were 1050 $^\circ$C for n-GaN, 650 $^\circ$C for InGaN/GaN QWs, and 990 $^\circ$C for the p-GaN and p$^+$-GaN layers.

The LED device was fabricated by standard binary photolithography and vacuum evaporation. We note that the shallow surface slopes of our 3D structures allows the additional planarization process, which is typically required for highly 3D structures^8,9,21^, to be skipped. The Ni (5 nm)/Au (10 nm) semi-transparent p-contact and Ti (50 nm)/Au (200 nm) p-pad electrodes were deposited on the sample surface. The dimensions of the p-contact electrodes were 50 $\times$ 50 $\mu$m$^2$ for Top and Slope A and 50 $\times$ 30 $\mu$m$^2$ for Slope B. In was used for n-electrodes. Here, each LED was not electrically isolated by etching the p-GaN layer. The device performance was assessed on-wafer at RT under DC injection. The emitted light was collected by an optical fiber.

### Fundamental characterization

The 3D shapes of the fabricated polyhedral structures were investigated by confocal laser scanning microscopy (KEYENCE, VK-9510). The off-angle profiles were analyzed using the cross-sectional profiles. Local emission properties of the InGaN QWs were evaluated by SEM and CL spectroscopy at RT (JEOL, JSM-6500F). The acceleration voltage was 5 kV.

### Supplementary Information

Supplementary Video Legend.

Supplementary Videos.

Supplementary Information.

## Data Availability

The datasets used and/or analyzed during the current study available from the corresponding author on reasonable request.
