# Supplementary material for: Flexible topographical design of light-emitting diodes realizing electrically controllable multi-wavelength spectra
Source: Sci Rep. 2023 Aug 4;13:12665. doi: 10.1038/s41598-023-39791-2 (PMC10403568; doi:10.1038/s41598-023-39791-2)
Supplement: Supplementary file 1 — Supplementary Video Legend. [file 41598_2023_39791_MOESM1_ESM.pdf]

The video showcases the operation of the monolithic multi-wavelength LED.
