# Supplementary material for: Flexible topographical design of light-emitting diodes realizing electrically controllable multi-wavelength spectra
Source: Sci Rep. 2023 Aug 4;13:12665. doi: 10.1038/s41598-023-39791-2 (PMC10403568; doi:10.1038/s41598-023-39791-2)
Supplement: Supplementary file 3 — Supplementary Information. [file 41598_2023_39791_MOESM3_ESM.pdf]

# SUPPLEMENTARY INFORMATION

**Flexible topographical design of light-emitting diodes realizing electrically controllable multi-wavelength spectra**

Yoshinobu Matsuda, Ryunosuke Umemoto, Mitsuru Funato, and Yoichi Kawakami

Department of Electronic Science and Engineering, Kyoto University,

Kyoto 615-8510, Japan

E-mail: yoshinobu.matsuda@optomater.kuee.kyoto-u.ac.jp, kawakami@kuee.kyoto-u.ac.jp

## Supplementary Note 1. Device process

Multi-wavelength light emitters based on three-dimensional (3D) structures formed by a selective area growth (SAG) technique often cause device process complexity compared to conventional monochromatic light emitters using planar structures [S1-S6]. An example of a 3D structure formed by SAG, as shown in Fig. S1(a), includes the steep side facets perpendicular to the substrate surface, which can lead to challenges such as poor electrode coverage. This issue may be overcome through

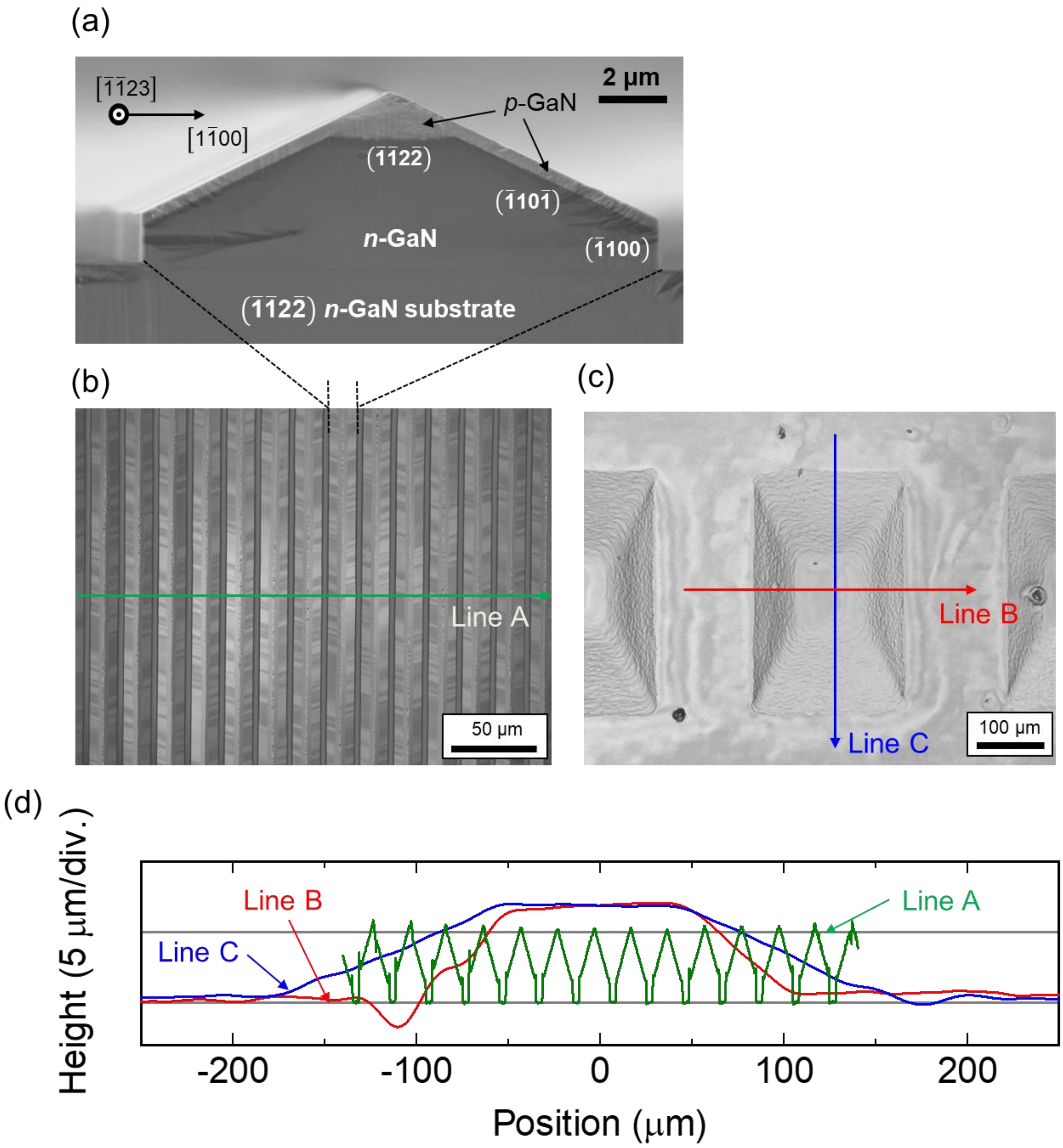


**Figure S1.** (a) Cross-sectional scanning electron microscopy image of a 3D LED structure on a $(\bar{1}\bar{1}2\bar{2})$ GaN substrate formed by SAG [S6]. Confocal laser scanning microscopy images of (b) the 3D LED structures by (b) SAG and (c) the proposed method. (d) Cross-sectional height profiles along the lines indicated in (b, c).

device process improvements but currently tends to hinder extracting the full performance of multi-wavelength light emitters. Therefore, it is significantly important to develop gently-sloping 3D structures with multi-wavelength emission properties. Figures S1(b) and S1(c) show confocal laser scanning microscopy images of the 3D light-emitting diode (LED) structures by SAG and the proposed method in this study, respectively. Cross-sectional height profiles along the lines indicated in Figs. S1(b) and S1(c) are presented in Fig. S1(d). Due to the shallow surface slopes of the proposed 3D structures, the same device processing as that for conventional planar LEDs can be employed. Furthermore, the process-friendly structures allow us to form individual electrodes on each part of the polyhedral structures without any additional effort.

## Supplementary Note 2. 3D shape evolution

In this study, we have developed a method to fabricate InGaN light-emitting diodes (LEDs) on polyhedral structures by using grayscale lithography, thermal reflow, reactive ion etching (RIE), and metal-organic vapor phase epitaxy (MOVPE). Grayscale lithography and thermal reflow processes define the polyhedral shape of the photoresist, and maintaining the three-dimensional (3D) shape during the subsequent processes is important. Figure S2 shows the 3D shapes after (a) grayscale lithography and thermal reflow, (b) RIE, and (c) MOVPE observed by confocal laser scanning microscopy. The respective height mapping images are shown in Figs. S2(d, e, f). Different from the main text, all the slopes of the polyhedral shape are congruent to simply confirm the shape evolution. The 3D shapes remain similar throughout all processes. On the other hand, the smooth surface obtained after grayscale lithography and RIE becomes rough after MOVPE. Although the off-angle variation caused by the roughness is not fatal for controlling the emission wavelengths from the slopes, the emission line widths of the overgrown InGaN LEDs increase due to the non-uniform emission wavelength distribution. The origin of the surface roughness is under investigation, but one possible reason is the non-uniform growth rate distribution during MOVPE due to the off-angle variation in the slopes after RIE. To mitigate this issue, further process optimization is required, such as developing methods to accurately define the 3D shapes before regrowth and optimizing growth conditions to prevent roughness formation.

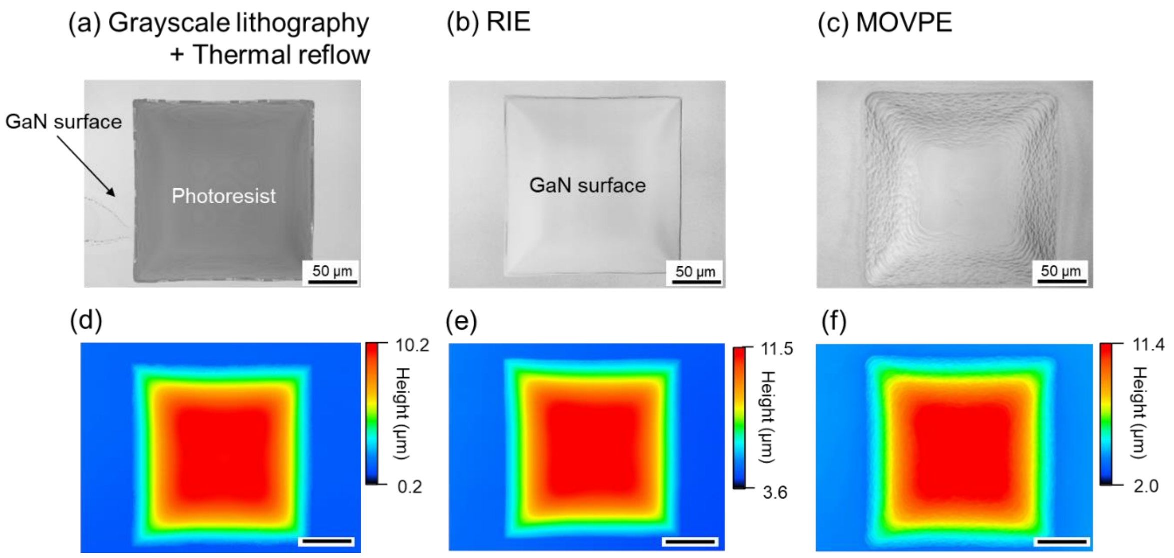


**Figure S2.** Confocal laser scanning microscopy images of the polyhedral shapes after (a) grayscale lithography and thermal reflow, (b) RIE, and (c) MOVPE, and (d, e, f) the respective height mapping images.

## Supplementary Note 3. QW structural analysis

Transmission electron microscopy (TEM) was used to investigate the structural properties of the InGaN quantum wells (QWs) regrown on the polyhedral structures. The local In compositions were estimated from energy dispersive X-ray spectroscopy (EDS) measurements under scanning TEM (STEM) mode. The method for evaluating the In compositions by EDS elemental analyses can be found in Ref. [S7]. Figure S3(a) shows a cross-sectional bright-field STEM image of the InGaN QW around the boundary between the slope and planar regions in the polyhedral structure. The estimated In compositions taken at the positions indicated in Fig. S3(a) are displayed in Fig. S3(b). The In composition at the planar region (position 6), corresponding to the (0001) plane, is much higher (around 40%) than those at the slope region (around 20%). On the other hand, typical high-resolution TEM (HR-TEM) images taken at the planar and slope regions in Fig. S3(a) are shown in Figs. S3(c) and S3(d), respectively. The InGaN well widths are the same, so the emission wavelength difference between the slope and planar regions is determined by the In composition distributions. In addition,

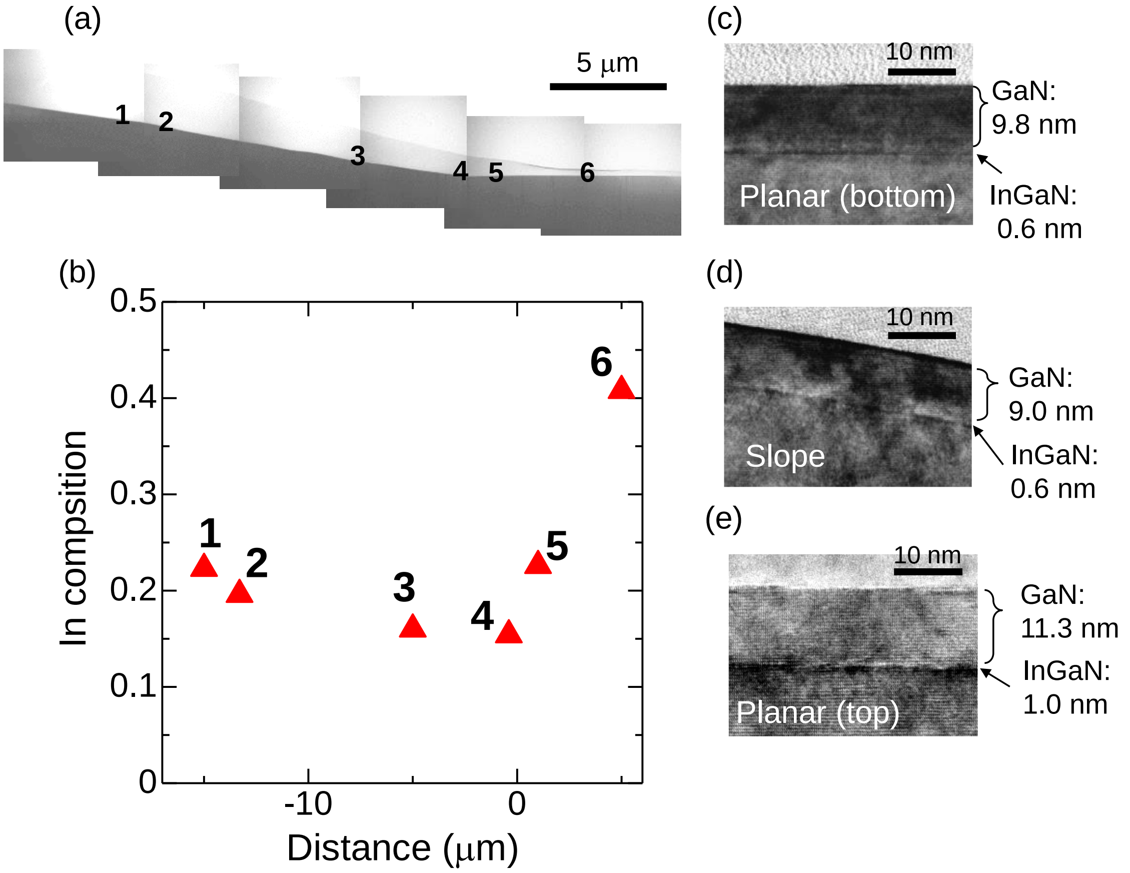


**Figure S3.** (a) Cross-sectional bright-field STEM image of the InGaN QW around the boundary between the slope and planar regions in the polyhedral structure. (b) Estimated In compositions at positions indicated in (a). Typical HR-TEM images at the (c) slope and (d) planar regions in (a) and (e) a planar region around the top of the polyhedral structure.

an HR-TEM image taken at a planar region around the top of the polyhedral structure is shown in Fig. S3(e). The InGaN well width is larger than that at the bottom planar region in Fig. S3(c). We confirmed that the emission peak wavelength at the bottom planar region is ~530 nm, slightly shorter than ~550 nm at the top planar region by local cathodoluminescence (CL) spectroscopy. This behavior is also suggested in Figs. 3(h) and 3(i) in the main text: the bottom planar region starts to emit prior to the top planar region with the longer monitoring wavelength. Therefore, the slight wavelength difference between them is attributed to the InGaN well widths.

## References


[S1] Kishino, K., Yanagihara, A., Ikeda, K., and Yamano, K. Monolithic Integration of Four-colour InGaN-based Nanocolumn LEDs. *Electron. Lett.* **51**, 852 (2015).

[S2] Schimpke, T., Mandl, M., Stoll, I., Pohl-Klein, B., Bichler, D., Zwaschka, F., Strube-Knyrim, J., Huckenbeck, B., Max, B., Müller, M., Veit, P., Bertram, F., Christen, J., Hartmann, J., Waag, A., Lugauer, H., Strassburg, M. Phosphor-Converted White Light from Blue-Emitting InGaN Microrod LEDs. *Physica Status Solidi A* **213**, 1577 (2016).

[S3] Schimpke, T., Lugauer, H. J., and Avramescu. A. Position-Controlled MOVPE Growth and Electro-Optical Characterization of Core-Shell InGaN/GaN Microrod LEDs. *Proc. SPIE* 9768, 97680T (2016).

[S4] Rishinaramangalam, A. K., Nami, M., Shima, D. M., Balakrishnan, G., Brueck, S. R. J., and Feezell, D. F. Reduction of Reverse-leakage Current in Selective-area-grown GaN-based Core–shell Nanostructure LEDs Using AlGaN Layers. *Physica Status Solidi A* **214**, 1600776 (2017).

[S5] Robin, Y., Bae, S. Y., Shubina, T. V., Pristovsek, M., Evropeitsev, E. A., Kirilenko, D. A., Davydov, V. Y., Smirnov, A. N., Toropov, A. A., Jmerik, V. N., Kushimoto, M., Nitta, S., Ivanov, S. V., and Amano, H. Insight into the Performance of Multi-Color InGaN/GaN Nanorod Light Emitting Diodes. *Sci. Rep.* **8**, 7311 (2018).

[S6] Matsuda, Y., Funato, M., and Kawakami, Y. Doping and Fabrication of Polar-Plane-Free Faceted InGaN LEDs with Polychromatic Emission Properties on $(\bar{1}\bar{1}2\bar{2})$ Semipolar Planes. *J. Appl. Phys.* **128**, 213103 (2020).

[S7] Nishizuka, K., Funato, M., Kawakami, Y., Fujita, S., Narukawa, Y., and Mukai, T. Efficient Radiative Recombination from $\langle 11\bar{2}2\rangle$-Oriented $In_xG_{1-x}N$ Multiple Quantum Wells Fabricated by the Regrowth Technique. *Appl. Phys. Lett.* **85**, 3122 (2004).
